# Refining Estimates of Bird Collision and Electrocution Mortality at Power Lines in the United States

**DOI:** 10.1371/journal.pone.0101565

**Published:** 2014-07-03

**Authors:** Scott R. Loss, Tom Will, Peter P. Marra

**Affiliations:** 1 Smithsonian Conservation Biology Institute – Migratory Bird Center, National Zoological Park, Washington, District of Columbia, United States of America; 2 Division of Migratory Birds – Midwest Regional Office, United States Fish and Wildlife Service, Bloomington, Minnesota, United States of America; University of Lleida, Spain

## Abstract

Collisions and electrocutions at power lines are thought to kill large numbers of birds in the United States annually. However, existing estimates of mortality are either speculative (for electrocution) or based on extrapolation of results from one study to all U.S. power lines (for collision). Because national-scale estimates of mortality and comparisons among threats are likely to be used for prioritizing policy and management strategies and for identifying major research needs, these estimates should be based on systematic and transparent assessment of rigorously collected data. We conducted a quantitative review that incorporated data from 14 studies meeting our inclusion criteria to estimate that between 12 and 64 million birds are killed each year at U.S. power lines, with between 8 and 57 million birds killed by collision and between 0.9 and 11.6 million birds killed by electrocution. Sensitivity analyses indicate that the majority of uncertainty in our estimates arises from variation in mortality rates across studies; this variation is due in part to the small sample of rigorously conducted studies that can be used to estimate mortality. Little information is available to quantify species-specific vulnerability to mortality at power lines; the available literature over-represents particular bird groups and habitats, and most studies only sample and present data for one or a few species. Furthermore, additional research is needed to clarify whether, to what degree, and in what regions populations of different bird species are affected by power line-related mortality. Nonetheless, our data-driven analysis suggests that the amount of bird mortality at U.S. power lines is substantial and that conservation management and policy is necessary to reduce this mortality.

## Introduction

Collisions and electrocutions of birds at power lines have long represented a major conservation issue [1], [2], and the current proliferation of electrical infrastructure is increasing this threat [3]. Globally, collisions with power lines may cause more than one billion annual bird deaths [4]. Between 10 and 41 million birds are likely killed each year by power line collisions in Canada [5]. In the United States, rough estimates of annual mortality range from hundreds of thousands to 175 million collisions [6], [7] and from tens to hundreds of thousands of electrocutions [7]. This amount of mortality would rank power lines above other structures that kill birds, including wind turbines and communication towers [8], [9]. Furthermore, mortality at power lines may contribute to population declines for some species, as evidenced by studies documenting that power line-caused mortality can cause a large percentage of total mortality for species from several avian orders [10]–[14].

Power line collisions occur when birds fly into wires; electrocutions occur at poles when a bird completes a circuit by touching two energized parts or an energized and grounded part [14], [15]. Correlates of mortality rates include: (1) biological factors (e.g., bird age, size, and wing span for both threats; maneuverability, flocking behavior, and vision for collision); (2) environmental factors (e.g., topography, vegetation, and prey abundance for both threats); and (3) structure-related factors (e.g. line orientation and distance between wires for both threats; exposure of and distance between energized and grounded parts for electrocution) [15]–[20]. Whereas electrocutions occur primarily at distribution lines–small power lines with voltages between 2.4 and 60 kilovolts (kV)–collisions occur at both distribution lines and transmission lines–large power lines with voltages >60 kV [16], [21], [22]. However, relatively few collision studies have been conducted at distribution lines; those that have suggest that there is little difference in collision rates between line types ([23]–[25] but see [20]). Both sources of mortality are reducible with the use of retrofitting measures [15], [19], [26]–[28] or with implementation of bird-safe standards at new construction [15], [16].

Despite an increasing number of studies that employ rigorous a priori study designs (e.g., [17], [21]), much of the research published to date about bird mortality at power lines has consisted of qualitative reviews and assessments of opportunistically collected data (hereafter “retrospective studies”) [22], [29]. Furthermore, nationwide estimates of mortality at U.S. power lines are speculative [7] or based on extrapolation from a single European study [6]. Policy and management for reduction of wildlife mortality should ideally be based on evidence from scientific studies that implement randomized and replicated sampling schemes (hereafter “prospective studies”). In addition, national-scale estimates of mortality and comparisons among mortality threats are likely to be used for prioritizing policy and management strategies and for identifying major research needs [30], [31]. These estimates should therefore be based on systematic and transparent assessment of rigorously collected data (e.g. [32]–[35]).

We conducted a systematic and quantitative review of U.S. and international studies that estimate mortality rates for bird collision and electrocution at power lines. To reduce bias in our estimates, we defined inclusion criteria by which studies were selected to ensure that only prospective and rigorously conducted studies were used in analyses. We quantified annual mortality and explicitly incorporated uncertainty by combining probability distributions of mortality rates, the amount of U.S. electrical infrastructure, and biases associated with carcass surveys. To highlight specific topics that require additional research, we also conducted sensitivity analyses to estimate how much uncertainty in our mortality estimates was contributed by each model component. Finally, we summarized the available species-specific data on bird collision fatalities at U.S. power lines.

## Materials and Methods

### Literature Search

**Figure 1:**
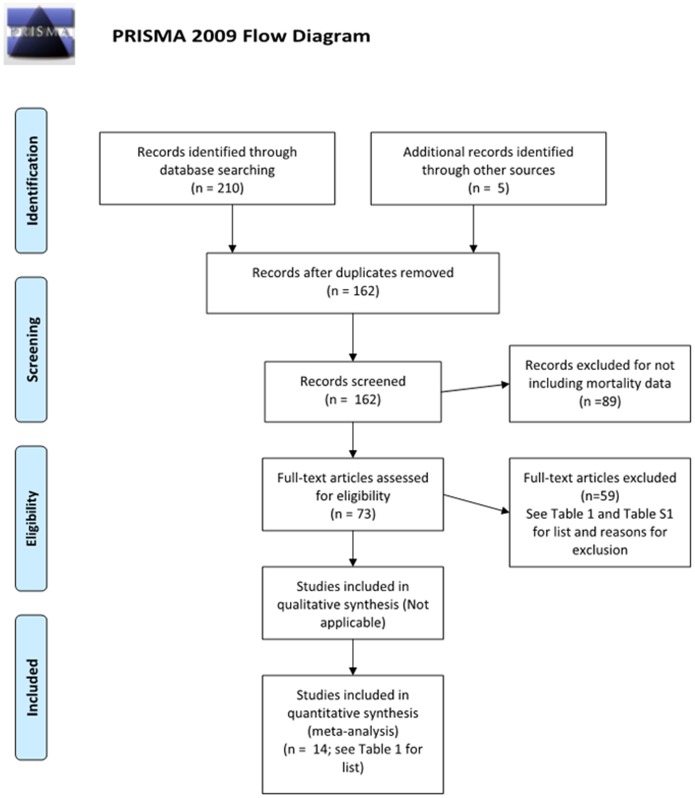
PRISMA flow diagram illustrating the number of independent articles and reports retrieved using the search strategy described under materials and methods, as well as the number of articles screened, excluded, and included in our systematic analysis of bird mortality from collision and electrocution at U.S. power lines.

We searched Google Scholar and the Web of Science database (using the Web of Knowledge search engine) to locate peer-reviewed articles and technical reports from U.S. and international studies of bird mortality at power lines. We also searched for studies providing estimates of the amount of U.S. electrical infrastructure (the number of power poles or length of power lines). We used the search terms: “bird electrocution,” “bird wire collision,” and “bird power line collision,” and the previous terms with “bird” replaced by “avian.” We also used: “United States length of electrical lines” and “United States number of electrical poles” and the previous two terms with “electrical” replaced by “power,” “distribution,” and “transmission;” “line” replaced by “wire;” and “pole” replaced by “pylon” and “tower.” We checked reference lists to locate additional sources, and we contacted three experts in the field to inquire if they knew of additional unpublished studies (R. Harness, R. Lehman, and R. Loughery, pers. comm.). Unlike studies of bird mortality at wind farms [9], [33] we located few industry reports that investigated mortality at power lines. Our analysis is therefore based on peer-reviewed studies, agency technical reports, and conference and workshop proceedings. We provide a flowchart illustrating the number of independent articles and reports retrieved using the above search strategy–as well as the number of articles screened, excluded, and included for our analysis of avian mortality–in Figure 1.

### Inclusion Criteria

To reduce variation among studies in sampling design and methodology and to minimize bias in our estimates, we implemented inclusion criteria for the studies used in our mortality estimates. To avoid duplication, we only included studies for in depth review if they presented data that had not been presented in earlier studies. For some studies, we included some data that met inclusion criteria and excluded other data that did not. Additional inclusion criteria were specified such that we excluded: (1) retrospective studies, (2) studies focusing only on a sub-set of bird groups, (3) studies that experimentally tested a retrofitting measure or included retrofitted lines without separately presenting data from retrofitted and control segments, (4) studies including but not separately reporting incidental records (i.e., records collected outside of standardized surveys), (5) studies not reporting the proportion of the calendar year covered by sampling and mortality rate estimates, (6) studies not reporting the extent of power line sampled (length of line or number of poles), (7) studies based on a single sampling occasion or on multiple sampling occasions covering less than one month (we arbitrarily selected a duration of one month to avoid including non-representative mortality rates that were exceptionally low or high), (8) studies of mortality from power lines and other threats (e.g., collisions with vehicles or wind turbines) not presenting data separately for each threat, and (9) studies of electrocution and collision not presenting data separately for each threat (this type of data would not allow separate estimation of collision and electrocution mortality rates).

### Data Extraction

We extracted a single mortality rate (as described in detail below: for collisions, number of carcasses per length of power line; for electrocutions, number of carcasses per pole) from each study meeting our criteria unless a study included both collision and electrocution data or data from both transmission and distribution lines. In these cases, we extracted rates separately for each data sub-set. We also extracted separate estimates when a single study included more than one non-adjacent sampling area or different study design and/or sampling methodologies during different time periods. Depending on the study, the extracted mortality rate was either an unadjusted count (i.e., not corrected for scavenger removal, imperfect searcher detection, or other survey-related biases) or a count that was adjusted for one or more of these sampling biases. As described briefly in in the following section and in further detail in Text S1, our final analysis only included unadjusted mortality rates, and we accounted for sampling biases in our mortality estimation model.

For collision and electrocution rate estimates, we standardized raw carcass counts by the length of power line and number of poles sampled, respectively. For studies greater than one year in duration that sampled a different amount of infrastructure each year, we calculated rates using the average amount of infrastructure sampled. For studies that were less than one year in duration, we accounted for the portion of the year not sampled in our estimation model described in the following section. For studies greater than one year in duration, we divided rates by the number of years of sampling or the fractional number of years sampled (e.g., 24 months = 2 years; 14 months = 1.17 years), thus assuming that mortality rates do not vary seasonally. Despite individual studies concluding that mortality rates can vary by season (e.g., [10], [18]), the vast majority of records meeting our inclusion criteria lacked dates of sampling, and the remaining records only listed the season of sampling. This limitation prevented us from closely examining seasonal variation in the extracted data.

In addition to extracting total mortality rate estimates, we also extracted raw species counts from U.S. studies that met criteria 1–4 above. Implementation of criteria 5 and 6 was unnecessary for generating unbiased species counts, and implementation of criteria 7–9 did not result in removal of additional studies beyond those removed by criteria 1–4. Because no electrocution studies met all criteria, we did not extract species data for this mortality source. All studies used for the mortality estimate and/or the species summary are illustrated in Table 1; excluded studies (along with reasons for exclusion) are in Table S1, and references for studies in Table S1, but not in the main manuscript, are in Text S2.

**Table 1:** Meta-data and mortality rates for studies meeting inclusion criteria for estimation of bird mortality at power lines in the United States and/or summary of mortality by species.

	Line type^a^		Sampling coverage	All species?^b^	Study used?		Mortality rate		
		Location			Total^c^	Species^d^	Per km	Per pole	Study
*Collision*									
U.S.									
	Trans	San Luis Valley, CO	Feb-Apr, Oct-Nov	Yes	Yes	Yes	1.05	?	[47]
	Trans	Dawson, ND	Apr-May; Jul-Nov	Yes	Yes	Yes	35.00	?	[48]
	Trans	Billings, MT	Mar-Oct	Yes	No^e^	Yes	480.30	?	[1]
	Trans	south-central NE	Mar-Apr	No	Yes	No	1.26	?	[56]
	Trans	southwest IN	Jan-Apr; Sept-Dec	Yes	No^f^	Yes	?	?	[45]
	Trans	Midland, MI	Mar-Dec	Yes	No^f^	Yes	?	?	[57]
	Trans	James Island, SC	Year Round	Yes	Yes	Yes	6.23	?	[58]
	Trans	south-central NE	Mar-Apr	No	No^e^	No	0.21	?	[49]
	Dist	San Luis Valley, CO	Feb-Apr, Oct-Nov	Yes	Yes	Yes	1.37	?	[47]
	Dist	Dawson, ND	Apr-May; Jul-Nov	Yes	Yes	Yes	7.14	?	[48]
	Dist	south-central NE	Mar-Apr	No	Yes	No	0.02	?	[49]
International									
	Trans	southwest Spain	Jan-Apr; Dec	Yes	Yes	No	2.27	?	[59]
	Trans	central Spain	Jan-May; Jul-Dec	Yes	No^g^	No	124.80	?	[27]
	Trans	south Norway	Year Round	No	Yes	No	3.70	?	[52] (section 1)
	Trans	central Spain	Year Round	No	No^g^	No	8.10	?	[60]
	Trans	North Wales	Apr-Jul	No	Yes	No	1.67	?	[61]
	Trans	central Spain	Year Round	Yes	Yes	No	2.89	?	[23] (section A)
	Trans	New S. Wales, Aust.	Jan-Mar; Nov-Dec	Yes	Yes	No	13.60	?	[62]
	Dist	central Spain	Jan-May; Jul-Dec	Yes	No^g^	No	99.00	?	[27]
	Dist	south Norway	Year Round	No	Yes	No	3.13	?	[52] (sec 2)
	Dist	south Norway	Year Round	No	Yes	No	6.53	?	[52] (sec 3)
	Dist	central Spain	Year Round	No	No^g^	No	2.70	?	[60]
	Dist	central Spain	Jan-Mar; Aug-Dec	Yes	Yes	No	8.72	?	[23] (sec B)
	Dist	central Spain	Year Round	Yes	Yes	No	14.17	?	[23] (sec C)
	?	north Greece	Year Round	No	Yes	No	14.12	2.40	[63]
	?	north-central India	Year Round	No	No^g^	No	0.26	?	[13]
*Electrocution*									
U.S.									
	Dist	northwest Colorado	Jun-Sept	No	Yes	No	?	0.022	[64]
	Dist	High Desert, CO/UT	Year Round	No	Yes	No	0.01	0.005	[65]
	Dist	Rangely, CO	Year Round	No	Yes	No	?	0.011	[65]
	Dist	Uintah, UT	Year Round	No	No^e^	No	0.02	0.036	[65]
	Dist	Owens Valley, CA	Jan-Apr; Nov-Dec	No	Yes	No	0.03	0.002	[66]
	Dist	San Jac. Valley, CA	Jan-Mar; Oct-Dec	No	Yes	No	0.02	0.001	[66]

a Type of power line studied, including low-voltage (2.4–60 kV) distribution lines (dist), high voltage (>60 kV) transmission lines (trans), both line types (both), or no information provided about line type (?).

b Does study survey for and present data for all bird species, not just particular species groups?

c Was study used in estimate of annual bird mortality at power lines?

d Was study used for species counts (Table S2)? Excluded U.S. studies focused on particular bird group(s) without including all species; all international studies were excluded because of different species assemblages in other countries).

e study meets inclusion criteria but was not included in the estimate of annual bird mortality because mortality rate is a statistical outlier among studies meeting inclusion criteria.

f study meets inclusion criteria for species summary but excluded from mortality estimate because no information provided about length of wire sampled.

g study meets inclusion criteria but was not included in the estimate of annual bird mortality because mortality rate is adjusted for biases associated with carcass surveys; these biases were later accounted for in our analyses (see main text).

### Relaxing Inclusion Criteria to Increase Sample Size

We felt that all inclusion criteria were necessary for producing the least biased mortality estimates possible; however, after implementing all criteria and extracting data, only 17 mortality rate estimates remained, including 15 collision rates (8 U.S. and 7 international) and 2 electrocution rates (0 U.S. and 2 international). We therefore examined whether sample sizes could be increased by relaxing some inclusion criteria that we considered less essential (criteria 2 and 7). Relaxation of criteria 2 (study must include all bird groups) resulted in inclusion of 19 additional rates, including 11 collision and 8 electrocution rates. Because of this large sample of additional data, we repeated collision mortality estimation with and without criteria 2 relaxed. For electrocution, the only studies meeting criteria 2 were international studies. Because we sought to avoid estimating electrocution mortality solely using international data, we estimated electrocution mortality only once using the U.S. studies accepted with criteria 2 relaxed. This approach likely contributes negative bias to our electrocution estimate because relaxing criteria 2 results in the inclusion of studies that do not sample all bird groups, and because all types of birds could potentially be killed by electrocution [15]. Relaxation of criteria 7 (study must not be based on a single short sampling occasion) resulted in inclusion of only two additional mortality rates; therefore, we did not repeat estimation with this criterion relaxed.

### Quantification of Annual Bird Mortality at Power Lines

When data from multiple independently conducted studies are combined to generate national estimates of annual mortality, the mortality rates should be standardized to account for the fact that different studies sample different proportions of the calendar year. Above, we described how we accounted for this variation in sampling coverage for studies that were greater than one year in duration. For studies that were less than one year in duration, the mortality rate estimates should ideally be standardized to year-round rates using year-round studies as a baseline [8], [34]. However, this type of standardization was not possible for our data set because there were few year-round studies that met our inclusion criteria and therefore few studies to use as a baseline for standardization. Among the year-round studies that did meet inclusion criteria, all electrocution studies and all but one collision study did not present data separately for different portions of the year, a limitation that prevented us from using this approach [34]. As described below, we therefore accounted for partial-year sampling coverage by applying a correction factor in the estimation model.

We estimated bird collision and electrocution mortality by multiplying data-derived probability distributions of mortality rates by distributions of the amount of infrastructure, and we also incorporated correction factors that account for biases associated with carcass surveys and partial-year sampling. We estimated collision mortality only for transmission lines because there is little bird collision data available for distribution lines and because there are no estimates for the length of distribution lines in the U.S. nor maps that would allow us to calculate this value (J. Goodrich-Mahoney, Electric Power Research Institute pers. comm.) (however, note that there are rough estimates of tens of millions of miles of distribution lines present in the U.S. [36], [37]). We estimated electrocution mortality only for distribution lines because electrocution is a greater concern at this power line type [22], [29], [38] and because all extracted electrocution data were from distribution lines. This approach likely contributes negative bias to our mortality estimates because both collisions and electrocutions can occur at both power line types (although, in general, there is relatively little evidence of widespread electrocution at transmission lines; but see 39). We used the following estimation model:
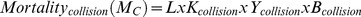
(1)

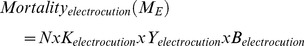
(2)

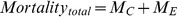
(3)where *L* is the length of transmission line corridors in the U.S.; *K* is the annual mortality rate per km of power line (collision) or per power pole (electrocution); *Y* is a correction factor that accounts for mortality occurring during portions of the year not covered by sampling in partial-year studies; *B* is a correction factor that accounts for four major biases: scavenger removal bias (under-estimation due to scavengers removing a proportion of carcasses between fatality surveys), searcher detection bias (under-estimation due to surveyors only detecting a proportion of the remaining carcasses), crippling bias (under-estimation due to a proportion of birds surviving long enough to exit the survey area before dying), and habitat bias (under-estimation due to a proportion of the survey area not being searchable to due dense vegetation, unsafe terrain, or other logistical constraints); and *N* is the number of distribution poles in the U.S. The partial-year correction (*Y*) was treated as a fixed value. From the uniform probability distribution defined for every other parameter (specific distributions in Table 2; rationale for distributions in Text S1), we drew a random value using the “runif” command in Program R and used the above formulas. We repeated this calculation 10,000 times to generate uncertainty bounds for estimates.

**Table 2:** Probability distributions used for estimation of bird mortality at power lines in the United States.

	Distribution	Distribution	
Parameter	type	parameters	Source
*Collision at transmission lines*			
Length of transmission lines (km)	Uniform	Min = 775,986; Max = 948,428	[36]; J. Goodrich-Mahoney pers. comm.
Mortality rate (per km) – all species	Uniform	Min = 2.91; Max = 15.57	95% C.I. across 10 studies meeting inclusion criteria
Mortality rate (per km) – focal species	Uniform	Min = 3.15; Max = 11.30	95% C.I. across 17 studies meeting inclusion criteria
Partial-year correction – all species	NA^a^	Estimate = 1.54	1/ave. proportion of year covered by studies in analysis
Partial-year correction – focal species	NA^a^	Estimate = 1.53	1/ave. proportion of year covered by studies in analysis
Bias correction factor	Uniform	Min = 1.25, Max = 3.28	Ave. ratio of adjusted to unadjusted mortality estimates
*Electrocution at distribution lines*			
Number of utility poles	Uniform	Min = 166.5 M; Max = 203.5 M	[67]
Mortality rate (per pole)	Uniform	Min = 0.001; Max = 0.016	95% C.I. across 5 studies meeting inclusion criteria
Partial-year sampling correction	NA^a^	Estimate = 1.5	1/ave. proportion of year covered by studies in analysis
Bias correction factor	Uniform	Min = 1.91, Max = 2.92	[68]

a Parameter is a point estimate, not a probability distribution.

### Sensitivity Analyses

Sensitivity analyses identified the contribution of each parameter to uncertainty in the mortality estimates. We defined multiple linear regression models, assumed a normal distribution of errors (function “lm” in Program R), treated mortality estimate replicates as values of the dependent variable, and treated randomly drawn values of each parameter as values of the independent variables. We interpreted the percentage of uncertainty explained by each parameter using partial r^2^ values [32], [34], [40], [41]. We repeated this analysis for the total mortality estimate (including all model parameters) and for the collision and electrocution estimates (including only the parameters from each respective sub-model).

### Counts of Bird Species Killed by Power Line Collisions

Six collision studies met inclusion criteria for the species summary. Of the records in these studies, 82.6% (*N* = 3,402) were identified to species (with remaining records identified to broader taxonomic groupings) and 78.1% were from a single study [1]. Given these limitations, we could not generate estimates of mortality by species [42], calculate vulnerability indices [43], or calculate average proportional representation of each species [34]. We therefore present raw counts of the bird species found in studies meeting our inclusion criteria (Table S2) and refrain from drawing conclusions about species-specific collision vulnerability.

## Results

All mortality estimates are summarized in Table 3. With inclusion criteria 2 relaxed (studies do not need to include all bird groups), we estimate annual U.S. bird mortality from power line collisions at between 7.7 and 42.4 million (median = 20.0 million). With inclusion criteria 2 enforced, we estimate annual collision mortality at between 8.0 and 57.3 million birds (median = 25.5 million). These estimates equate to median annual collision rates of 23.2 birds/km of power line (95% CI = 8.9–49.2) and 29.6 birds/km of power line (95% CI = 9.3–66.4), with inclusion criteria 2 relaxed and enforced, respectively. We estimate that between 0.9 and 11.6 million birds (median = 5.6 million) are electrocuted each year at U.S. distribution lines. This equates to a median annual rate of 0.03 birds per distribution pole (95% CI = 0.005–0.062). Combining both threats, we estimate total annual power-line caused mortality at between 11.8 and 49.2 million birds (median = 25.9 million) with inclusion criteria 2 relaxed and between 12.6 and 64.0 million birds (median = 31.2 million) with criteria 2 enforced.

**Table 3:** Estimates of annual bird mortality at U.S. power lines.

	Mean units of U.S. infrastructure	Total mortality (millions)		Mortality per km/pole	
Mortality type		Median	95% CI	Median	95% CI
Collision at transmission lines	862,207 km	25.48 M^a^	7.98–57.25 M^a^	29.6^a^	9.3–66.4^a^
		20.01 M^b^	7.67–42.43 M^b^	23.2^b^	8.9–49.2^b^
Electrocution at distribution lines	185 M poles	5.63 M	0.92–11.55 M	0.030	0.005–0.062
TOTAL		31.16 M^a^	12.63–63.98 M^a^		
		25.85 M^b^	11.84–49.28 M^b^		

a Estimate based on enforcing study inclusion criteria that mortality surveys must survey and present data for all bird species.

b Estimate based on relaxing study inclusion criteria that mortality surveys must survey and present data for all bird species.

Due to the relatively large amount of mortality caused by collisions and variable collision rates across studies, the collision mortality rate parameter explained the greatest percentage of uncertainty in our estimates of collision mortality (65.6%) and total power line-related mortality (62.4%). For the collision estimate, almost all remaining uncertainty (26.8%) was explained by the bias correction factor. Other factors explaining at least 5% of uncertainty in the total estimate included the bias correction factor for collision mortality (25.4%) and the electrocution rate (5.0%). Due to variable electrocution rates across studies, the electrocution rate parameter explained the majority of uncertainty in the electrocution estimate (91.9%).

Raw species counts are shown in Table S2. These results are descriptive of the studies that met our inclusion criteria, but this data set contains substantial sampling bias. All six studies were at power lines that crossed or were in close proximity to water bodies. The 19 species with the highest counts–and 36 of the 42 species recorded–are waterbirds. All land birds, including raptors, were counted 16 or fewer total times as collision casualties.

## Discussion

Our annual estimates of between 8 and 57 million birds killed by collision and between 0.9 and 11.6 million birds killed by electrocution indicate that bird mortality at U.S. power lines constitutes a major source of anthropogenic mortality. The range of our estimates for power lines is greater than systematically derived U.S. estimates for all other anthropogenic structural threats except buildings (365–988 million [41]), including collisions with communication towers (6.6 million [8]), collisions with all wind turbines (573,000 [9]), and collisions with modern mono-pole wind turbines (140,000–328,000 [33]). National estimates of anthropogenic mortality and comparisons of different mortality sources can be useful for prioritizing conservation policies [30], [31]. Our estimates in particular should alert conservation biologists and policy-makers to the continued problem of bird mortality caused by power lines. Furthermore, our sensitivity analyses highlight major research gaps that need to be addressed in order to increase understanding of this issue and therefore to advance mitigation efforts.

### Comparison to other mortality estimates

Our estimate range for power line collisions falls within the much broader range of previous figures that are either speculative (hundreds of thousands to 175 million [7]) or based on extrapolation of results from a single study to all U.S. transmission lines (130 million [6]). We improved upon earlier collision estimates by systematically incorporating data from 11 U.S. and international studies, including 17 mortality rate estimates. Our estimated range of between 0.9 and 11.6 million birds electrocuted annually is based on systematic analysis of five unique mortality rate estimates and is greater than the only other estimate to date, a speculative figure of tens to hundreds of thousands of birds [7]. As expected, the collision mortality estimate generated from studies that included all bird groups was higher than the estimate that included studies focused on particular species. Birds of all sizes and taxonomic orders collide with power lines [15], [44], [45], and collision studies that only include large species (e.g. waterbirds, raptors, and/or game birds) likely under-estimate total mortality rates. Consideration of our higher collision estimate (between 8 and 57 million birds) would be appropriate under a precautionary approach to mortality management [46].

The above figures could be underestimates because we did not calculate collision mortality at distribution lines or electrocution mortality at transmission lines and because both types of mortality occur. Collision studies at distribution lines report mortality rates between 0.02 and 7.14 birds/km [47]–[49], and some studies suggest that there is little difference in collision rates between the two line types [23]–[25]. Few studies have documented electrocution at transmission lines; however, raptor electrocution rates in Arizona were found to be the same at both line types [39]. Estimation of collision mortality at distribution lines would require speculation about the length of U.S. distribution lines, and estimation of electrocution mortality at transmission lines would require speculation about electrocution rates at this line type. Because a central objective of our study was to conduct data-driven analyses, we did not generate these estimates.

The lack of data about which bird species are killed, and how the species composition of fatalities varies across habitats, prevented us from quantitatively estimating vulnerability of different species to mortality at power lines. The species count for power line collisions is biased towards water birds because all studies meeting inclusion criteria for this analysis were at or near bodies of water. For electrocution, the vast majority (91.7%) of fatality records from studies used to estimate mortality were raptors. Our electrocution estimate could therefore be viewed as a rough approximation of the number of annual raptor electrocutions in the U.S. However, identifying which raptor species experience disproportionately high electrocution risk is not possible, given the small sample (n = 132) of total raptor records across the studies we used. A qualitative literature appraisal indicates that eagles dominate the reported electrocution records [22], and that the Golden Eagle in particular (*Aquila chrysaetos*) may experience the greatest electrocution risk due to a combination of its large body size and preference for open habitats without natural perches [11], [18]. Eagles were not well-represented in our quantitative analysis because most eagle fatalities are documented as isolated incidents or from retrospective band-recovery or radio-tracking studies that did not meet our inclusion criteria.

### Research Needs and Estimate Limitations

Parameters that explain a large proportion of uncertainty in our estimates can be inferred to indicate major research gaps that, if addressed, will improve understanding of power line-related mortality and assist mitigation efforts [34], [41]. A large proportion of uncertainty in our estimates was explained by highly variable mortality rates that led us to define broad probability distributions. This finding indicates that additional replication of collision and electrocution studies that meet the standards of rigor embodied by our inclusion criteria are needed to further increase precision of mortality estimates. Research is especially needed in under-represented regions and habitat types; electrocution studies have focused disproportionately on the western U.S. and collision studies have focused disproportionately on wetlands. The most useful data will be collected in prospective studies that base sampling on randomization and replication, that sample all groups of birds, and that sample during all months of the year. In our comprehensive review of the literature, we found no mortality rate estimates that fulfilled all of these standards.

The bias correction factor for collisions also explained substantial uncertainty in our estimates. This finding suggests that additional research is needed to quantify how bias sources (scavenger removal, imperfect carcass detection, crippling, and habitat bias) cause raw counts to under-estimate mortality. Most collision rates that we extracted (76% of U.S. rates) were not corrected for any of the above biases. Recent research into bird and bat collisions at wind facilities provides an example of how quantitative methods that account for these biases can be developed and applied [9], [50].

Sampling design and data collection methods varied among the studies we used, and we were unable to account for all of these differences. Nonetheless, we accounted for substantial methodological variation by implementing inclusion criteria, by applying a correction factor to account for studies sampling varying proportions of the year, and by standardizing raw carcass counts by the amount of infrastructure sampled. A limitation of our estimate is that although most studies attempted to confirm whether birds had been killed by collision or electrocution, there may have been some error associated with designating the specific cause of death. Some apparent collision victims (e.g. those found under the middle of a wire span) may have been electrocuted by touching two wires, and some apparent electrocution victims may have been electrocuted when colliding with wires [51]. This potential error source may have led to positive or negative estimation bias in individual studies; however, our approach of developing probability distributions using multiple studies likely reduced the effect of this within-study bias. Finally, positive bias could have been contributed to our estimates by only including data from power lines with no retrofitting measures in place. An unknown proportion of U.S. power lines likely have reduced mortality rates due to retrofitting measures.

We were unable to quantify seasonal patterns of mortality due to a limited sample of studies that surveyed year-round and a limited number of records that included date information. Several nuances related to seasonal bird movements and life histories likely influence seasonal patterns of mortality risk at power lines. First, migratory birds may be more vulnerable to collisions at transmission lines during spring and fall migration periods because birds move at higher altitudes during migration than they do during sedentary periods. The opposite is also likely to be true; resident bird species (and migratory species during sedentary periods) are likely more vulnerable to collisions at distribution lines because flights during these periods tend to occur at relatively low altitudes. Second, many locations are characterized by drastically different local bird communities during different periods of the year, and high latitude areas have particularly large fluctuations in species diversity due to seasonal movements of migratory species. This seasonal variation affects the pool of species that are at risk of experiencing collision or electrocution mortality. Finally, some species (especially gallinaceous birds – family Phasianidae) experience the greatest risk of collision mortality during winter as a result of poor lighting and weather conditions [10], [52]. Given the above complexities, additional year-round studies are necessary to improve understanding of seasonal variation in mortality at power lines.

Perhaps more than other mortality sources, studies of bird collision and electrocution mortality at power lines tend to focus on areas that are already known to experience bird deaths. These mortality hotspots include power lines near large populations of birds or high quality habitat. For electrocutions, power poles in flat landscapes without trees are especially attractive to birds as perches, are associated with a greater risk of collision, and have received the greatest amount of study [22]. We sought to minimize the bias contributed by non-random sampling and spatial clustering by excluding mortality rates from our analyses that were high statistical outliers. Nonetheless, the predisposition to study mortality hotspots, and the observation that in many regions a relatively small fraction of poles cause electrocutions [17], [21], suggests that extrapolating published mortality rates across the U.S. power grid could contribute positive bias to national mortality estimates. Non-random sampling of power lines also leads to a biased representation of bird species composition, as evidenced by our species summary. Documentation of high mortality rates at “problem” lines is crucial for implementing mitigation measures to reduce mortality. However, future studies that aim to produce unbiased estimates of mortality rates should also employ random sampling designs that sample multiple habitat and power line types without regard to a priori expectations. This random sampling structure allows more accurate estimation of mortality rates, identification of mortality correlates, extrapolation of mortality rates to larger scales, and assessment of species-specific risks.

### Conclusions

Collisions and electrocutions at U.S. power lines represent a major source of bird mortality. Because a proportion of this mortality is preventable, policies and management measures should be implemented whenever possible to reduce the number of bird deaths incurred. The most cost-effective approach to reducing power line-related mortality will likely be to implement bird-friendly design strategies at new power lines (see “best practices” in [15], [16]). However, mortality reduction is also possible with retrofitting of existing lines. For collision mortality, retrofitting measures include marking of wires and removing ground wires. For electrocution mortality, measures include capping energized parts and increasing spacing between energized parts and grounded parts [15], [16]. Notably, there has been increasing use of steel distribution poles in the U.S. [38], and due to increased conductivity of electricity, these poles can lead to particularly high rates of electrocution [28]. Mitigation measures for steel poles are different than those for wooden poles (see [16], [28]), and it will be particularly important to implement these steps to reduce bird electrocution mortality in the U.S. and internationally.

Mortality monitoring should also be conducted to ensure that design and retro-fitting measures achieve desired mortality reductions. APLIC guidelines have resulted in substantial advances in addressing bird mortality at power lines. However, there is still little information available to assess the proportion of U.S. infrastructure with bird-friendly designs or retrofitting measures in place or the degree to which such measures reduce mortality [22]. There is also no consistent and peer-reviewed monitoring protocol to assess bird mortality at power lines. A national mortality reporting database can facilitate standardization of data collection and management for mortality monitoring at power lines and for other threats [46]. In addition to mandatory monitoring and reporting under U.S. Fish and Wildlife Service (USFWS) permits, there is currently a voluntary injury and mortality reporting system maintained by the USFWS (the Bird Incident Mortality Reporting System). Roughly 40 U.S. electrical utilities currently report mortality data to this system (A.M. Manville II, pers. comm.).

Linking specific mortality causes to population level impacts is exceptionally difficult in the absence of large samples of species-specific mortality data and comprehensive population monitoring information [53]. Given the above-discussed deficiencies in species-specific data, national-scale population impacts of power line-related bird mortality remain unclear. Nevertheless, some regions and bird species could experience significant population level impacts, as suggested by U.S. studies indicating that power lines cause a large proportion of mortality for some species, primarily raptors [11], [18]. National mortality estimates will be most useful when also complemented by fine-scale intensive research that allows for assessment of population responses to mortality (e.g., [54], [55]) and for the development of targeted management objectives. Nonetheless, the absence of a clear link between mortality at power lines and population impacts should not prevent mortality reduction measures from being taken, especially given imperfect understanding about how multiple mortality threats interact to cumulatively impact wildlife populations [46].

## Supporting Information

Table S1 Studies reviewed but excluded from analyses.
(DOCX)

Table S2 Raw counts of bird species found in studies of power line collision.
(DOCX)

Text S1 **Supplementary Methods.** Detailed description of methods used to define probability distributions in mortality estimation model.
(DOCX)

Text S2 **References in Supplementary Information.** References for literature cited in the supporting information (Text S1 and Table S1), but not in the main text.
(DOCX)
