# Supplementary material for: Refining Estimates of Bird Collision and Electrocution Mortality at Power Lines in the United States
Source: PLoS One. 2014 Jul 3;9(7):e101565. doi: 10.1371/journal.pone.0101565 (PMC4081594; doi:10.1371/journal.pone.0101565)
Supplement: Table S1 — Studies reviewed but excluded from analyses. (DOCX) [file pone.0101565.s001.docx]

**Table S1. Studies reviewed but excluded from analyses**.

|  | Line type^a^ | | Location | Reason for exclusion^b^ | Study |
| --- | --- | --- | --- | --- | --- |
| *Collision* |  | |  |  |  |
| U.S. |  | |  |  |  |
|  | Trans | | Bismarck, ND | *8* | [71] |
|  | Trans | | central ND | *2,6* | [73] |
|  | Both | | south-central CO | *4* | [44] |
|  | ? | | southeast ID | *1,2* | [74] |
|  | ? | | US and Canada | *1,2* | [75] |
|  | ? | | northwest OK, east NM | *1,2* | [76] |
| International |  | |  |  |  |
|  | Trans | | south Norway | *2,10* | [77 (Sec 1)] |
|  | Trans | | south Norway | *2,10* | [77 (Sec 2)] |
|  | Trans | | south Norway | *2,3* | [77 (Sec 3)] |
|  | Trans | | south Norway | *2,10* | [77 (Sec 4)] |
|  | Trans | | southwest Africa/Namibia | *2,5* | [78] |
|  | Trans | | Colombia | *3,5* | [79] |
|  | Trans | | United Kingdom | *3,5* | [80] |
|  | Trans | | Lomellina, Italy | *5* | [81] |
|  | Both | | southwest Spain | *2,10* | [72] |
|  | Both | | northeast Venezuela | *7* | [82] |
|  | Both | | Overberg, South Africa | *2,3* | [24] |
|  | ? | | central Spain | *10* | [83] |
|  | ? | | Italy | *5* | [81] |
|  |  | |  |  |  |
| *Electrocution* |  | |  |  |  |
| U.S. |  | |  |  |  |
|  | Dist | | western US | *1,2* | [84] |
|  | Dist | | western US | *1,2* | [18] |
|  | Dist | | northwest CO | *1,2,3* | [85] |
|  | Dist | | Altamont Pass, CA | *1,2* | [86] |
|  | Both | | Tucson, AZ | *4* | [39] |
|  | Both | | Tucson, AZ | *2,4* | [87] |
|  | ? | | ID, OR, WY, UT, NV, NM | *2,5* | [88] |
|  | ? | | Chesapeake Bay, MD/VA | *1,2* | [89] |
|  | ? | | Altamont Pass, CA | *1,2* | [90] |
|  | ? | | north FL | *1,2,8* | [91] |
|  | ? | | 24 US States | *1,2* | [92] |
|  | ? | | 26 US States | *1,2* | [93] |
|  | ? | | Denali, AK | *1,2* | [11] |
|  | ? | | west-central FL | *1,2* | [94] |
|  | ? | | north CA | *1,2* | [95] |
|  | ? | | Flathead Reservation, MT | *2,8* | [96] |
|  | ? | | Montana | *1* | [97] |
|  | ? | | Idaho | *1,2* | [98] |
|  | ? | | western US | *1,2* | [99] |
|  | ? | | Arizona | *1,2* | [100] |
| International |  | |  |  |  |
|  | Dist | | Russia | *11* | [101] |
|  | Dist | | Italy | *5* | [81] |
|  | ? | | Sudan, East Africa | *2,7* | [102] |
|  | ? | | Canary Islands | *2,7* | [103] |
|  | ? | | southwest Spain | *11* | [70] |
|  | ? | | central Spain | *11* | [60] |
|  | ? | | southwest Spain | *10* | [83] |
|  | ? | | northeast Spain | *11* | [104] |
|  | ? | | south England | *1,2* | [105] |
|  | ? | | northeast Spain | *5* | [19] |
|  |  | |  |  |  |
| *Mortality cause not identified* | | | |  |  |
|  | | Trans | Spain | *1,2,9* | [106] |
|  | | ? | Cape Province, South Africa | *1,2,9* | [107] |
|  | | ? | Spain | *1,2,9* | [108] |
|  | | ? | ID, MT, WY | *1,2,9* | [109] |
|  | | ? | Montana | *1,2,9* | [110] |
|  | | ? | central Kazakhstan | *9* | [111] |
|  | | ? | Europe | *1,2,9* | [112] |
|  | | ? | Aberdeen, MD | *1,2,9* | [113] |
|  | | ? | Spain | *1,2,9* | [14] |
|  | | ? | AZ, south CA, Baja California | *1,2,9* | [114] |
|  | | ? | north Italy | *1,2,9* | [115] |
|  | |  |  |  |  |

^a^Type of power line studied, including low-voltage (2.4-60 kV) distribution lines (dist), high voltage (>60 kV) transmission lines (trans), both line types (both), or no information provided about line type (?)

^b^Numbers correspond to inclusion criteria described in main text (except *10* and *11* which are described but not numbered in the main text): *1* **-** Study lacks a prospective sampling component (i.e. based on retro-spective sampling of fatalities); *2* - Study focuses on a particular species group (e.g. raptors) and did not search for all bird species; *3* – Study experimentally tests a retrofitting measure or includes retrofitted lines without presenting data from retrofitted and control segments separately; *4* - Study is prospective but was excluded because it includes records collected incidentally (i.e. outside of standardized surveys); *5* - Study does not provide information about the proportion of the calendar year covered by sampling; *6* - Study does not provide the amount of power line sampled (length of line or number of poles; *7* - Study is based on one or more sampling occasions covering less than one month total; *8* - Study documents mortality from electrical infrastructure and other anthropogenic threats (e.g. collision with fences/vehicles), but does not present data separately for different mortality sources; *9* - Study documents mortality from both collisions and electrocution, but does not present data separately for different mortality sources; *10* - Study includes data overlapping that presented in another study; *11* - Study met all inclusion criteria, but later removed because included large sample of metal utility poles
