# Supplementary material for: Refining Estimates of Bird Collision and Electrocution Mortality at Power Lines in the United States
Source: PLoS One. 2014 Jul 3;9(7):e101565. doi: 10.1371/journal.pone.0101565 (PMC4081594; doi:10.1371/journal.pone.0101565)
Supplement: Table S2 — Raw counts of bird species found in studies of power line collision. (DOCX) [file pone.0101565.s002.docx]

**Table S2.** **Raw counts of bird species found in studies of power line collision.** Total raw counts of bird species found as collision fatalities in studies of U.S. transmission power lines meeting inclusion criteria for species summary (see main text for specific criteria) [1, 45, 47, 48, 57, 58].

|  | Total | Number of facilities |
| --- | --- | --- |
| Species | count | with fatalities |
| Eared Grebe | 920 | 1 |
| Blue-winged Teal | 439 | 2 |
| American Coot | 278 | 4 |
| Northern Pintail | 264 | 1 |
| Green-winged Teal | 213 | 2 |
| Wilson's Phalarope | 208 | 1 |
| Gadwall | 185 | 2 |
| Lesser Yellowlegs | 146 | 3 |
| Sandhill Crane | 140 | 2 |
| Mallard | 122 | 4 |
| Northern Shoveler | 84 | 2 |
| Ruddy Duck | 48 | 2 |
| Amerian Avocet | 43 | 1 |
| American Wigeon | 42 | 1 |
| Ring-billed Gull | 31 | 2 |
| Greater Yellowlegs | 29 | 1 |
| Redhead | 24 | 1 |
| Canada Goose | 18 | 2 |
| Baird's Sandpiper | 16 | 1 |
| Yellow-headed Blackbird | 16 | 1 |
| Herring Gull | 15 | 1 |
| Great Blue Heron | 14 | 3 |
| Stilt Sandpiper | 14 | 1 |
| Sora | 13 | 2 |
| Pied-billed Grebe | 12 | 1 |
| American White Pelican | 10 | 1 |
| Franklin's Gull | 10 | 1 |
| Great Egret | 10 | 1 |
| Killdeer | 10 | 2 |
| California Gull | 5 | 1 |
| Double-crested Cormorant | 4 | 1 |
| American Black Duck | 3 | 1 |
| Red-winged Blackbird | 3 | 1 |
| Whooping Crane | 3 | 1 |
| Black-crowned Night Heron | 2 | 2 |
| Yellow-billed Cuckoo | 2 | 1 |
| Common Snipe | 1 | 1 |
| Horned Lark | 1 | 1 |
| Northern Harrier | 1 | 1 |
| Rock Pigeon | 1 | 1 |
| Sanderling | 1 | 1 |
| Wood Duck | 1 | 1 |
| Unknown Gull | 167 | 4 |
| Unknown Waterfowl | 166 | 1 |
| Unknown Bird | 99 | 3 |
| Unknown Shorebird | 54 | 2 |
| Unknown Rail | 46 | 1 |
| Unknown Cormorant | 40 | 1 |
| Unknown Passerine | 30 | 1 |
| Unknown Blackbird | 26 | 1 |
| Unknown Sandpiper | 24 | 1 |
| Unknown Grebe | 12 | 1 |
| Unknown Grouse | 11 | 1 |
| Unknown Wading Bird | 11 | 1 |
| Unknown Dove | 10 | 2 |
| Unknown Raptor | 10 | 1 |
| Unknown Duck | 5 | 1 |
| Unknown Heron | 3 | 1 |
| Unknown Woodpecker | 3 | 1 |
