# Supplementary material for: Refining Estimates of Bird Collision and Electrocution Mortality at Power Lines in the United States
Source: PLoS One. 2014 Jul 3;9(7):e101565. doi: 10.1371/journal.pone.0101565 (PMC4081594; doi:10.1371/journal.pone.0101565)
Supplement: Text S1 — Supplementary Methods. Detailed description of methods used to define probability distributions in mortality estimation model. (DOCX) [file pone.0101565.s003.docx]

**Text S1. Supplementary Methods**. Detailed description of methods used to define probability distributions in mortality estimation model

We defined probability distributions for each of the model parameters described in the main text except for the partial-year sampling correction factor (*Y*), which we treated as a fixed value. We defined uniform probability distributions for parameters because there was not enough data to ascribe higher probability to any particular value in the defined range. In some cases where relatively little data was available to define lower and/or upper bounds to distributions, we defined arbitrary, albeit plausible, values. We examined the effect of defining these ranges in the overall sensitivity analysis (see main text), and we also present selected results from this analysis below. We argue that incorporation of plausible values into the estimation model—and formally quantifying sensitivity of mortality estimates to parameter values—is preferable to not quantifying mortality at all and instead relying on current non-quantitative estimates.

**Length of transmission line corridors in the U.S. (*L*)** Several estimates for the length of U.S. transmission line corridors exist; however, most do not cite the source of the original estimate. Some estimates only pertain to transmission lines with very high voltages (e.g. >320,000 km of lines >230 kV [69]); however, transmission lines are usually defined (including in our analysis) to include all lines with voltages greater than 60 kV [16]. An estimate of 862,207 km of transmission line was cited in [36] with attribution to a personal communication. We contacted the cited source and confirmed that the estimate was for all transmission lines with voltage >60 kV and that it was generated based on analysis of plat maps and published in a contractor report. The estimate reflects “ground km” of transmission line corridor (i.e. not wire length, which is often greater than corridor length) and is the latest and most accurate estimate (J. Goodrich-Mahoney, Electrical Power Research Institute, pers. comm.). We therefore used 862,207 as a mean value, incorporated estimate uncertainty by assuming ±10% error around this value, and defined a uniform distribution with this uncertainty range (775,986 – 948,428 km). Admittedly, the range of uncertainty around the point estimate is arbitrary; however, no additional data exists to quantitatively estimate the uncertainty associated with this parameter. Sensitivity analysis indicated that the range of values we used for this parameter contributed <1% of total uncertainty for both the collision mortality estimate and the estimate of total mortality at power lines. Therefore, if the true length of transmission line corridor in the U.S. is actually lower or higher than the above range, then our mortality estimate is only slightly over-estimated or under-estimated, respectively.

**Number of distribution line poles in the U.S. (*N*)** The latest estimate for the number of distribution poles that we are aware of is 185 million poles [67], a figure that has been cited for at least eight years [22]. Because we could not found a more recent estimate, we used this figure as the mean value of the probability distribution, incorporated estimate uncertainty by assuming ±10% error, and defined a uniform distribution with this uncertainty range (166.5 – 203.5 million). As with transmission line length, the uncertainty bounds for this parameter were defined arbitrarily, but the parameter contributed a small amount of variance to mortality estimates (1% for the electrocution estimate: 0.2% for the total estimate). Our mortality estimate would therefore be only slightly over-estimated or under-estimated if the true number of poles was lower or higher, respectively, than the above parameter range.

**Annual mortality rate (*K*).** For both collision and electrocution, we compiled mortality rate estimates and used box plots to identify and remove statistical outliers (see Table 1 for values removed). For collision mortality, we used both U.S. and international studies to develop probability distributions because there were relatively few (7) U.S. collision rates remaining after implementation of inclusion criteria and removal of outliers. Of the remaining international studies, five presented mortality rates that were adjusted for one or more biases (e.g. scavenger removal). Because most collision rate estimates were not adjusted for biases, we excluded the adjusted estimates from development of probability distributions (Table 1) and accounted for biases using the correction factor described below. This resulted in 10 remaining international collision rates that we used for analysis. For electrocution, only two international studies remained after removing outliers. Because these studies focused on metal poles [60, 70] and because wooden poles are the predominant pole type in the U.S., we only used U.S. mortality rates (*N*=5) to generate the electrocution rate probability distribution. As with collisions, none of these electrocution studies were adjusted for biases, and we applied a correction factor to account for this as described below.

From the remaining mortality rate estimates, we calculated the 95% confidence interval across studies for both collision and electrocution and used the lower and upper bounds of each interval to specify minimum and maximum values of uniform distributions. For collision mortality, we generated one probability distribution using the set of studies with inclusion criteria 2 relaxed (*N*_U.S._ = 7; *N*_international_ = 10; *N*_TOTAL_ = 17) and one probability distribution using the sub-set of studies with criteria 2 enforced (*N*_U.S._ = 5; *N*_international_ = 5; *N*_TOTAL_ = 10).

**Partial-year sampling correction factor (*Y*).** For collision and electrocution estimates, we incorporated a fixed-value correction factor to account for sampling and mortality rates only covering a portion of the year. This correction factor was different for each of the separate mortality estimates (collision mortality, criteria 2 relaxed; collision mortality, criteria 2 enforced; electrocution mortality). We first calculated the average proportion of the year covered by the set of studies used to generate each mortality rate probability distribution. The correction factors were then generated by using the inverse of this proportion (e.g. if average proportion of the year sampled = 0.667, then correction factor = 1/0.667 = 1.5). To assess the validity of this approach, we individually corrected each partial-year study according to the proportion of the year sampled, and we re-calculated mortality rate probability distributions. The adjusted mortality rate distributions generated in this manner were nearly identical to the distributions generated by multiplying unadjusted mortality distributions by the partial-year correction factor. As discussed in further detail in the methods section, this approach assumes no seasonal variation in mortality rates, an assumption that could not be tested with the available mortality data.

**Bias correction factor (*B*).** For collision mortality, we applied a correction factor to adjust for the biases most often accounted for in studies of collision at power lines: scavenger removal bias (under-estimation due to scavengers removing a proportion of carcasses between fatality surveys), searcher detection bias (under-estimation due to surveyors only detecting a proportion of the remaining carcasses), crippling bias (under-estimation due to a proportion of birds surviving long enough to exit the survey area before dying), and habitat bias (under-estimation due to a proportion of the survey area not being searchable to due dense vegetation, unsafe terrain, or other logistical constraints). Four studies in our data set, including six separate mortality rate estimates, included both unadjusted mortality rates and rates adjusted for the above four biases [48, 58, 71, 72]. Across the six sets of estimates, the average factor of increase from the unadjusted to adjusted estimate was 2.19 (min = 1.25; max = 3.28). We used this minimum and maximum to define a uniform distribution for the collision correction factor.

For electrocution, no studies provided both unadjusted estimates and estimates adjusted for all four biases. We therefore used results from the only study to date designed to assess scavenger removal and searcher detection biases at power lines [68]. This study, conducted in an agricultural setting in Europe, derives a logarithmic model to predict the cumulative percentage of carcasses removed by scavengers from underneath power lines, as calculated from data generated during carcass removal trials that lasted 28 days. The study also estimates searcher detection probability; however, it does not estimate crippling bias or habitat bias. We therefore did not account for the latter two biases in our electrocution estimate.

We estimated a minimum and maximum scavenger removal percentage using the logarithmic model illustrated in figure 3 of [68], which estimates removal percentages for each day after carcasses are placed (we used the model for the large size class of carcasses because the majority of our electrocution records were raptors or other large species). The average length of the search interval for the five studies used in our electrocution estimate was 67.5 days. Entering this value into the logarithmic model yields an estimate of 52.3% of carcasses removed by scavengers. We considered this to be a maximum removal percentage because extrapolating beyond the bounds of the 28 day period of the field trials may result in unrealistically high removal values. To generate a minimum removal percentage, we calculated the estimated percentage of carcasses removed after 28 days (41.8%). In [68], the authors also present results of searcher detection probability trials. They found that 71.7% of large carcasses (pheasants) were detected by searchers. The majority of electrocution fatalities in the studies used in our mortality estimate were raptors and corvids. To allow for the possibility that these species are more detectable than pheasants (i.e. due to larger size and either darker or more contrasting plumage for some species), we considered 71.7% to be a minimum estimate of detection probability and arbitrarily considered 90% as a maximum detection probability.

Drawing on the above values, we generated minimum and maximum bias correction factors (correction factor = 1 / [proportion of carcasses not removed x proportion of carcasses detected]), and we defined the bounds of a uniform distribution using these values. For the maximum estimate of the correction factor, we used the higher scavenger removal estimate based on the 67.5 day period (47.7% of carcasses not removed) and the lower detection probability of 71.7% (1/0.477/0.717 = 2.92). For the minimum estimate of the correction factor, we used the lower scavenger removal estimate based on the 28 day period (58.2% of carcasses not removed) and the higher detection probability of 90% (1/.582/.900 = 1.909). Admittedly, the upper value of detection probability for electrocution surveys was arbitrarily defined due to a lack of available quantitative data; however, the bias correction factor for electrocution only contributed 5.7% of variance to the electrocution estimate and 0.1% of variance to the total mortality estimate. Therefore, deviations of the detection probability from the range of values we defined would be expected to contribute only minimal estimation bias relative to the magnitude of our mortality estimates.
