# Supplementary material for: Refining Estimates of Bird Collision and Electrocution Mortality at Power Lines in the United States
Source: PLoS One. 2014 Jul 3;9(7):e101565. doi: 10.1371/journal.pone.0101565 (PMC4081594; doi:10.1371/journal.pone.0101565)
Supplement: Text S2 — References in Supplementary Information. References for literature cited in the supporting information (Text S1 and Table S1), but not in the main text. (DOCX) [file pone.0101565.s004.docx]

**Text S2. References in Supplementary Information** – References for literature cited in the supporting information (Text S1, Table S1, and/or Table S2) but not in the main text. References 1-68 are listed in main text.

1. Edison Electric Institute (2013) Electricity Transmission. Available Online: http://www.eei.org/OURISSUES/ELECTRICITYTRANSMISSION/Pages/default.aspx (Accessed 3-23-14).
2. Ferrer M, de la Riva M, Castroviejo J (1991) Electrocution of Raptors on Power Lines in Southwestern Spain. J Field Ornithol 62: 181-190.
3. Electric Power Research Institute (EPRI) (2003) Bird Strike Indicator/Bird Activity Monitor and Field Assessment of Avian Fatalities. Interim Report 1005385; EPRI, Palo Alto, CA; Audubon National Wildlife Refuge, Coleharbor, ND; Edison Electric Institute, Washington, DC; Bonneville Power Administration, Portland, OR; California Energy Commission, Sacramento, CA; North Western Energy, Butte, MT; Otter Tail Power Company, Fergus Falls, MN; Southern California Edison, Rosemead, CA; Western Area Power Administration, Lakewood, CO.
4. Janss GF, Ferrer M (2000) Common crane and great bustard collision with power lines: collision rate and risk exposure. Wildl Soc Bull 28: 675-680.
5. Faanes CA, Johnson DH (1992) Cranes and power lines: an analysis of the issue. In: Wood DA, editor. Proceedings of the 1988 North American Crane Workshop. State of Florida Game and Fresh Water Fish Commission Nongame Wildlife Progam Technical Report No. 12. pp 297-312.
6. Beck JL, Reese KP, Connelly JW, Lucia MB (2006) Movements and survival of juvenile greater sage-grouse in southeastern Idaho. Wildl Soc Bull 34: 1070-1078.
7. Lewis JC, Kuyt E, Schwindt KE, Stehn TV (1992) Mortality in fledged Whooping cranes of the Arkansas/Wood Buffalo Population. In: Wood DA, editor. Proceedings of the 1988 North American Crane Workshop. State of Florida Game and Fresh Water Fish Commission Nongame Wildlife Progam Technical Report No. 12. pp 145-148.
8. Wolfe DH, Patten MA, Shochat E, Pruett CL, Sherrod SK (2007) Causes and patterns of mortality in lesser prairie-chickens (*Tympanuchus pallidicinctus*) and implications for management. Wildl Biol 13: 95-104.
9. Bevanger K, Brøseth H (2004) Impact of power lines on bird mortality in a subalpine area. Animal Biodiv Conserv 27: 67-77.
10. Brown CJ, Lawson JL (1989) Birds and electricity transmission lines in South West Africa/Namibia. Madoqua 16: 59-67.
11. De La Zerda S, Rosselli L (2002) Mitigating Collision of Birds Against Transmission Lines in Wetland Areas in Colombia, by Marking the Ground Wire with Bird Flight Diverters (BFD). In: 7th International Symposium on Environmental Concerns in Rights-of-Way Management. pp 395-401.
12. Scott RE, Roberts LJ, Cadbury CJ (1972) Bird deaths from power lines at Dungeness. British Birds 65: 273–286.
13. Rubolini D, Gustin M, Bogliani G, Garavaglia R (2005) Birds and powerlines in Italy: an assessment. Bird Conserv Int 15: 131-145.
14. McNeil R, Rodriguez JR, Ouellet H (1985) Bird mortality at a power transmission line in northeastern Venezuela. Biol Conserv 31: 153-165.
15. Janss GFE (2000) Avian mortality from power lines: a morphologic approach of a species-specific mortality. Biol Conserv 95: 353–359.
16. Harness RE, Wilson KR (2000) Raptor Electrocutions and Outages - A Review of Rural Utility Records spanning 1986-1996. In: Chancellor RD, Meyburg BU, editors Raptors at risk: proceedings of the Fifth World Conference on Birds of Prey and Owls. World Working Group on Birds of Prey, Berlin, Germany, and Hancock House Publishers, Surrey, British Columbia, Canada. pp 765-772.
17. Harness RE (2001b) The effectiveness of perch guards to prevent raptor electrocutions. In Carlton RG, editor. Avian Interactions with Utility and Communication Structures, Proceedings of a workshop in Charleston, SC December 2-3, 1999. pp 87-99.
18. Hunt WG (1998) A Population Study of Golden Eagles in the Altamont Pass Wind Resource Area: Population Trend Analysis 1994-1997--Executive Summary. In: Proceedings of National Avian - Wind Power Planning Meeting III, San Diego, California, May 1998. Prepared for the Avian Subcommittee of the National Wind Coordinating Committee by LGL Ltd., King City, Ontario. pp 15-17.
19. Dwyer JF, Mannan RW (2009) Return rates of aluminum versus plastic leg bands from electrocuted Harris's Hawks (*Parabuteo unicinctus*). J Raptor Res 43: 152-154.
20. Benson PC (1980) Large raptor electrocution and power pole utilization: a study in six western states. In Howard RP, Gore JF, editors. Proceedings of a Workshop on Raptors and Energy Developments. Idaho Chapter, The Wildlife Society, Boise, ID. pp 34-40.
21. Buehler DA, Fraiser JD, Seeger JKD, Therres GD, Byrd MA (1991) Survival rates and populations dynamics of bald eagles on Chesapeake Bay. J Wildl Manage 55: 608-613.
22. California Energy Commission (1989) Avian mortality at large wind energy facilities in California: identification of a problem. California Energy Commission, Sacramento, California, USA. Available from the US Geological Survey, Richard R. Olendorff Memorial Library, 970 Lusk St. Boise, Idaho 83706, USA.
23. Deem SL, Terrell SP, Forrester DJ (1998) A retrospective study of morbidity and mortality of raptors in Florida, 1988-1994. J Zool Wildl Med 29: 160-164.
24. Franson JC, Little SE (1996) Diagnostic findings in 132 great horned owls. J Raptor Res 30: 1-6.
25. Franson JC, Thomas NJ, Smith MR, Robbins AH, Newman S, et al. (1996) A retrospective study of postmortem findings in red-tailed hawks. J Raptor Res 30: 7-14.
26. Millsap B, Breen T, McConnell E, Steffer T, Phillips L, et al. (2004) Comparative fecundity and survival of bald eagles fledged from suburban and rural natal area in Florida. J Wildl Manage 68: 1018-1031.
27. Morishita TY, Fullerton AT, Lowenstine LJ, Gardner IA, Brooks DL (1998) Morbidity and mortality in free-living raptorial birds of northern California: a retrospective study, 1983-1994. J Avian Med Surg 12: 78-81.
28. Olson CV (2002) Human-related causes of raptor mortality in western Montana: things are not always as they seem. In: Carlton RG, editor. Proceedings: Avian Interactions with Utility and Communication Structures, International Workshop, Electric Power Research Institute, Palo Alto, California, USA. pp 71-82.
29. O'Neil T (1988) An analysis of bird electrocutions in Montana. J Raptor Res 22: 27-28.
30. Peacock E (1980) Powerline Electrocution of Raptors. In: Howard RP, Gore JF, editors. Workshop on Raptors and Energy Developments: Proceedings of a workshop held in Boise, Idaho January 25-26, 1980. pp 2-5.
31. Phillips RL (1986) Current issues concerning the management of golden eagles in western USA. In: Chancellor RD, Meyburg B-U, editors. Birds of Prey Bulletin No. 3. Proceedings of the Western Hemisphere Meeting of the World Working Group on Birds of Prey, Berlin, Germany. pp 149-156.
32. Whaley WH (1986) Population ecology of the Harris’ hawk in Arizona. Raptor Res 20: 1-15.
33. Matsina AI (2005) The estimation and prediction of killed raptors by electrocutions on the power lines in the Nizhniy Novgorod District (forest and forest-steppe zones of the center of the European part of Russia). Raptors Conserv 2: 33-41.
34. Angelov I, Hashim I, Oppel S (2012) Persistent electrocution mortality of Egyptian Vultures *Neophron percnopterus* over 28 years in East Africa. Bird Conserv Int 23: 1-6.
35. Donázar JA, Palacios CJ, Gangoso L, Ceballos, O, Gonzalez MJ et al. (2002) Conservation status and limiting factors in the endangered population of Egyptian vulture (*Neophron percnopterus*) in the Canary Islands. Biol Conserv 107: 89-97.
36. Mañosa S (2001) Strategies to identify dangerous electricity pylons. Biodiv Conserv 10: 1997–2012.
37. Meek WR, Burman PJ, Nowakowski M, Sparks TH, Burman NJ (2003) Barn owl release in lowland southern England—a twenty-one year study. Biol Conserv 109: 271-282.
38. Mañosa S, Real J (2001) Potential negative effects of collisions with transmission lines on a Bonelli's Eagle population. J Raptor Res 35: 247-252.
39. Boshoff AF, Minnie JC, Tambling CJ, Michael MD (2011) The impact of power line-related mortality on the Cape Vulture *(Gyps coprotheres*) in a part of its range, with an emphasis on electrocution. Bird Conserv Int 21: 311-327.
40. Gonzalez LM, Margalida A, Manosa S, Sanchez R, Oria J, et al. (2007) Causes and spatio-temporal variations of non-natural mortality in the vulnerable Spanish imperial eagle (*Aquila adalberti*) during a recovery period. Oryz 41: 495-502.
41. Harmata AR, Montopoli GJ, Oakleaf B, Harmata PJ, Restani M (1999) Movements and survival of bald eagles banded in the Greater Yellowstone Ecosystem. J Wildl Manage 63: 781-793.
42. Harmata AR, Restania M, Montpoli GJ, Zelenek JR, Ensign JT (2001) Movements and mortality of ferruginous hawks banded in Montana. J Field Ornithol 72: 389-378.
43. Lasch US, Zerbe S, Lenk M (2010) Electrocution of raptors at power lines in Central Kazakhstan. Raptor Conserv 18: 35-45.
44. Margalida A, Heredia R, Razin M, Hernandez M (2008) Sources of variation in mortality of the Bearded vulture (*Gypaetus barbatus*) in Europe. Bird Conserv Int 18: 1-10.
45. Mojica EK, Watts BD, Paul JT, Voss ST, Pottie J (2009) Factors contributing to bald eagle electrocutions and line collisions on Aberdeen Proving Ground, Maryland. J Raptor Res 43: 57-61.
46. Rideout BA, Stalis I, Papendick R, Pessier A, Puschner B et al. (2012) Patterns of Mortality in Free-ranging California Condors (*Gymnogyps californiaus*). J Wildl Dis 48: 95-112.
47. Rubolini D, Bassi E, Bogliani G, Galeotti P, Garvavaglia R (2001) Eagle Owl (*Bubo bubo*) and power line interactions in the Italian Alps. Bird Conserv Int 11: 319-324.
